# Engaging young adult clients of community pharmacies for HIV screening in Coastal Kenya: a cross-sectional study

**DOI:** 10.1136/sextrans-2014-051751

**Published:** 2014-12-08

**Authors:** Peter M Mugo, Henrieke A B Prins, Elizabeth W Wahome, Grace M Mwashigadi, Alexander N Thiong'o, Evanson Gichuru, Anisa Omar, Susan M Graham, Eduard J Sanders

**Affiliations:** 1 Kenya Medical Research Institute, Kilifi, Kenya; 2 Ministry of Health, Kilifi, Kenya; 3 University of Washington, Seattle, USA; 4 Oxford University, Headington, UK; 5 University of Amsterdam, Amsterdam, The Netherlands

**Keywords:** HIV TESTING, REFERRAL, PRIMARY CARE, DEVELOPING WORLD, HEALTH SERV RESEARCH

## Abstract

**Background:**

Adults in developing countries frequently use community pharmacies as the first and often only source of care. The objective of this study was to assess the success of pharmacy referrals and uptake of HIV testing by young adult clients of community pharmacies in the context of a screening programme for acute HIV-1 infection (AHI).

**Methods:**

We requested five pharmacies to refer clients meeting predefined criteria (ie, 18–29 years of age and requesting treatment for fever, diarrhoea, sexually transmitted infection (STI) symptoms or body pains) for HIV-1 testing and AHI screening at selected clinics. Using multivariable logistical regression, we determined client characteristics associated with HIV-1 test uptake.

**Results:**

From February through July 2013, 1490 pharmacy clients met targeting criteria (range of weekly averages across pharmacies: 4–35). Of these, 1074 (72%) accepted a referral coupon, 377 (25%) reported at a study clinic, 353 (24%) were HIV-1 tested and 127 (9%) met criteria for the AHI study. Of those tested, 14 (4.0%) were HIV-1 infected. Test uptake varied significantly by referring pharmacy and was higher for clients who presented at the pharmacy without a prescription versus those with a prescription, and for clients who sought care for STI symptoms.

**Conclusions:**

About a quarter of targeted pharmacy clients took up HIV-1 testing. Clients seeking care directly at the pharmacy (ie, without a prescription) and those with STI symptoms were more likely to take up HIV-1 testing. Engagement of adult pharmacy clients for HIV-1 screening may identify undiagnosed individuals and offers opportunities for HIV-1 prevention research.

## Introduction

Adults in developing countries frequently use community pharmacies as the first and often only source of care, owing to greater accessibility, lower cost or greater perceived privacy.^1^ ^2^ The factors driving direct care seeking at pharmacies may not be easily modifiable, yet pharmacy clients have not been included in provider-initiated testing and counselling programmes^3^ ^4^ or targeted in community-based strategies.^5^ In an assessment of treatment practices, we found that only 10% pharmacies would recommend HIV-1 testing to a client with urethritis.^6^

We have recently reported results of an acute HIV infection (AHI) screening study based in clinics, with referral of potentially eligible participants from pharmacies.^7^ The objective for the present study was to evaluate the success of pharmacy referrals and uptake of HIV testing by pharmacy clients in the context of this AHI screening programme.

## Methods

### Selection and training of pharmacies

The study was conducted in Mtwapa area of Coastal Kenya. Out of 20 pharmacies included in a previous survey,^6^ we selected five that had reported high client loads in the survey and were willing to refer clients for research. We trained pharmacy staff on client engagement and referral through frequent on-site visits (at least weekly) and occasional off-site sessions (beginning of the study and at least monthly). Pharmacies provided input to the design of study materials, including a leaflet of key messages and a referral coupon.

### Client referral procedure

Pharmacy workers were requested to target all clients aged 18–29 years purchasing medicine for fever, sexually transmitted infection (STI) symptoms, diarrhoea or body pains. These clients were referred using a numbered coupon to any of five participating clinics for free HIV-1 testing and screening for the AHI study. Other testing and follow-up procedures are detailed elsewhere.^7^ Client engagement was supported by three community volunteers. Pharmacy staff received Ksh 125 (US$1≈KSh 85) when a client presented at a study clinic and an additional Ksh 75 if the client was enrolled in the AHI study.

### Data collection and analysis

Data collected at the pharmacy included age, sex, whether client had a prescription, area of residence, symptom for which treatment was sought (recording multiple symptoms when reported), referring pharmacy, staff initials and referral date. Characteristics captured were of the presenting client, unless the client self-identified as a surrogate client (eg, partner), in which case details of the actual patient were captured. We compared the sex variable across pharmacy and clinical data, as a rough measure to assess whether the coupon changed hands.

Factors associated with test uptake (number tested/number targeted) were examined by univariable and multivariable logistical regression. A sensitivity analysis was conducted to evaluate factors associated with test uptake among the subset of pharmacy clients who accepted the referral coupon. All variables with p<0.10 from univariable modelling were included in multivariable modelling. A two-sided p value <0.05 indicated statistical significance. All analyses were performed using Stata (StataCorp, College Station, Texas, USA).

## Results

From February through July 2013, 1490 clients met AHI targeting criteria (range of weekly averages across pharmacies: 4–35). Of these, 1074 (72.1%) accepted a referral coupon, 377 (25%) reported for screening at study clinics, 353 (24%) were HIV-1 tested (21 were unwilling to test and three were known HIV positive) and 127 (9%) met criteria for the AHI study. Of those tested, 43 (13%) had never tested before, 52 (15%) had not tested in the last year and 14 (4.0%) were HIV-1 infected.

Test uptake varied significantly by referring pharmacy and was higher for clients who presented at the pharmacy without a prescription versus those with a prescription (table 1). Seeking treatment for STI symptoms was marginally associated with higher test uptake, while body pains were associated with lower test uptake. Among the subset of clients accepting the referral coupon, test uptake was higher for those without a prescription (aOR 2.3, 95% CI 1.5 to 3.6, p<0.001) and those with STI symptoms (aOR 1.3, 95% CI 1.0 to 2.0, p=0.03).

**Table 1:** Characteristics of adult pharmacy clients referred and tested for HIV-1 in Coastal Kenya, 2013

Characteristic	Targeted, n	Tested n (% of targeted)	Univariable analysis		Multivariable analysis**	
			OR (95% CI)	p Value	OR (95% CI)	p Value
Total number of clients	1490	353 (24%)	n/a	n/a		
Referring pharmacy						
Pharmacy 1	262	22 (8%)	Ref	Ref	Ref	Ref
Pharmacy 2	96	16 (17%)	2.2 (1.1 to 4.4)	0.03	2.2 (1.1 to 4.4)	0.03
Pharmacy 3	380	74 (19%)	2.6 (1.5 to 4.4)	<0.001	1.9 (1.1 to 3.2)	0.02
Pharmacy 4	60	12 (20%)	2.7 (1.3 to 5.9)	0.01	2.1 (1.0 to 4.6)	0.06
Pharmacy 5	692	229 (33%)	5.3 (3.4 to 8.6)	<0.001	5.0 (3.1 to 8.1)	<0.001
Age, years						
18–24	681	150 (22%)	Ref	Ref	–	–
25–29	809	203 (25%)	1.2 (0.9 to 1.5)	0.2	–	–
Sex of person presenting at pharmacy*						
Female	706	168 (24%)	Ref	Ref	–	–
Male	760	184 (24%)	1.0 (0.8 to 1.3)	0.9	–	–
Area of residence†						
Shanzu	145	27 (19%)	Ref	Ref	–	–
Mtwapa	1333	316 (24%)	1.4 (0.9 to 2.1)	0.2	–	–
Prescription presented‡						
Yes	361	29 (8%)	Ref	Ref	Ref	Ref
No	1109	318 (29%)	4.6 (3.1 to 6.9)	<0.001	5.0 (3.3 to 7.7)	<0.001
Treatment sought§						
Fever						
No	748	178 (24%)	Ref	Ref	–	–
Yes	742	175 (24%)	1.0 (0.8 to 1.3)	0.9	–	–
Diarrhoea						
No	1288	309 (24%)	Ref	Ref	–	–
Yes	202	44 (22%)	0.9 (0.6 to 1.3)	0.5	–	–
STI symptoms						
No	1135	237 (21%)	Ref	Ref	Ref	Ref
Yes	355	116 (33%)	1.8 (1.4 to 2.4)	<0.001	1.3 (1.0 to 1.8)	0.08
Body pains						
No	953	256 (27%)	Ref	Ref	Ref	Ref
Yes	537	97 (18%)	0.5 (0.7 to 0.8)	<0.001	0.5 (0.3 to 0.6)	<0.001

*Sex was missing for 24 clients. For 96 referrals, the sex of the person presenting at the study clinic was different from that of the person initially referred, mainly due to sharing of referral coupons.

**Variables included in the multivariable analysis were: referring pharmacy, prescription presented (yes/no), STI symptoms (yes/no) and body pains (yes/no).

†Area of residence was missing for 12 clients.‡Prescription was missing for 20 clients.§A total of 339 (22.7%) clients sought treatment for more than one symptom, including 333 with two symptoms and six with three symptoms. Antimalarial medications were counted as treatment for fever.

The odds of an HIV diagnosis was higher among clients aged 25–29 years than those aged 18–24 years (aOR 10.5, 95% CI 1.3 to 82.2, p=0.03), but did not vary by pharmacy, sex, residence, prescription or treatment sought.

## Discussion

To our knowledge, this is the first study in a developing country setting to target community pharmacy clients for HIV-1 testing and engagement in prevention research. Over 70% of targeted clients accepted the referral coupon; a quarter took the test and over a quarter of those tested had not tested in the last year. Clients seeking care directly at the pharmacy (ie, without a prescription) and those with STI symptoms were more likely to take up testing.

Our findings demonstrate that pharmacy clients can be engaged for HIV-1 testing and prevention research. However, further research is needed to identify factors associated with HIV test uptake at pharmacies in non-research settings. Results of this pilot study suggest that referral uptake could be boosted through improved communication skills among pharmacy workers and by focusing on patients with STI symptoms.

Alternatives to referral also need to be explored. We documented demand for HIV testing kits at some of the participating pharmacies, although anecdotally. Previous studies have found self-testing to be feasible and effective in community settings,^8^ and Kenyan National Guidelines recognise the role of the community pharmacies as a reliable source of self-testing kits.^9^ A self-testing intervention should be developed, targeting clients most at-risk for undiagnosed prevalent HIV-1 (eg, patients presenting with STI symptoms or fever). Such an intervention could increase awareness of HIV status and uptake of recommended retesting among Kenyan adults.^9^ Testing could also be done within the pharmacy premises for patients who are illiterate or uncomfortable testing themselves. The feasibility of such an approach has been proven in the USA^10^ and should be explored in our setting.

This study has limitations, including selection of pharmacies with documented high numbers of clients, a narrow age group (ie, young adults aged 18–29 years), a short study period; possible sharing of coupons, and the research context of this assessment, since patients being screened for the AHI study as well as pharmacy staff received financial incentives.

In conclusion, we found that engagement of adult pharmacy clients for HIV-1 testing may identify undiagnosed individuals and may offer opportunities for prevention research. Clients seeking care directly at the pharmacy without a prescription and those with STI symptoms were more likely to take up HIV-1 testing. Operational research is required to identify ways to improve HIV test access and uptake among pharmacy clients.

**Key messages**

- Targeting community pharmacy clients for HIV-1 testing resulted in detection of undiagnosed infections and enhanced knowledge of HIV status.
- Engagement of pharmacy clients in HIV-1 prevention research is feasible.
- HIV-1 testing at pharmacy premises or provision of self-testing services through pharmacies may increase HIV testing and diagnosis, and should be assessed in developing country settings.
